# Sulfite Alters the Mitochondrial Network in Molybdenum Cofactor Deficiency

**DOI:** 10.3389/fgene.2020.594828

**Published:** 2021-01-07

**Authors:** Anna-Theresa Mellis, Juliane Roeper, Albert L. Misko, Joshua Kohl, Guenter Schwarz

**Affiliations:** ^1^Department of Chemistry, Institute for Biochemistry, University of Cologne, Cologne, Germany; ^2^Department of Neurology, Massachusetts General Hospital, Harvard Medical School, Boston, MA, United States; ^3^Center for Molecular Medicine, University of Cologne, Cologne, Germany

**Keywords:** sulfite, sulfite oxidase, mitochondria, molybdenum cofactor deficiency, fission and fusion

## Abstract

Molybdenum cofactor deficiency (MoCD) is an autosomal recessive disorder belonging to the large family of inborn errors in metabolism. Patients typically present with encephalopathy and seizures early after birth and develop severe neurodegeneration within the first few weeks of life. The main pathomechanism underlying MoCD is the loss of function of sulfite oxidase (SO), a molybdenum cofactor (Moco) dependent enzyme located in mitochondrial intermembrane space. SO catalyzes the oxidation of sulfite (SO_3_^2–^) to sulfate (SO_4_^2–^) in the terminal reaction of cysteine catabolism, and in the absence of its activity, sulfurous compounds such as SO_3_^2–^, S-sulfocysteine, and thiosulfate accumulate in patients. Despite growing evidence that these compounds affect neuronal and mitochondrial function, the molecular basis of neuronal dysfunction and cell death in MoCD is still poorly understood. Here we show that mitochondria are severely affected by the loss of SO activity. SO-deficient mouse embryonic fibroblasts display reduced growth rates and impaired ATP production when cultured in galactose, which is an indicator of mitochondrial dysfunction. We also found that mitochondria in SO-deficient cells form a highly interconnected network compared to controls while displaying a slight decrease in motility and unchanged mitochondrial mass. Moreover, we show that the mitochondrial network is directly influenced by SO_3_^2–^, as a moderate elevation of SO_3_^2–^ lead to the formation of an interconnected mitochondrial network, while high SO_3_^2–^ levels induced fragmentation. Finally, we found a highly interconnected mitochondrial network in MoCD patient-derived fibroblasts, similar to our findings in mouse-derived fibroblasts. We therefore conclude that altered mitochondrial dynamics are an important contributor to the disease phenotype and suggest that MoCD should be included among the mitochondrial disorders.

## Introduction

Mitochondrial sulfite oxidase (SO) catalyzes the terminal step in the catabolism of cysteine and methionine, the oxidation of toxic sulfite (SO_3_^2–^) to sulfate (SO_4_^2–^), thereby passing two electrons to cytochrome *c* (Johnson and Rajagopalan, 1979). The cellular concentration of cysteine is tightly controlled by its synthesis and degradation (Stipanuk and Ueki, 2011), the latter proceeding through two distinct pathways. The first, often referred to as the oxidative pathway, yields taurine, and SO_4_^2–^ as the main end products (Kohl et al., 2018). The second pathway involves enzymatic reactions that regulate the formation and clearance of H_2_S, an important signaling molecule in mammals, and terminates in the production of thiosulfate and SO_4_^2–^. SO_3_^2–^ is a common metabolic intermediate in both catabolic pathways, that, if not removed by SO, can lead to various toxic effects (Vincent et al., 2004; Zhang et al., 2004; de Moura Alvorcem et al., 2017).

In man, the inherited loss of SO activity is caused by two different genetic mechanisms. First, SO may be impaired by mutations in the *SUOX* gene, thus leading to isolated sulfite oxidase deficiency (ISOD). Second, SO may be compromised by mutations in the molybdenum cofactor (Moco) biosynthetic genes (*MOCS1, MOCS2, MOCS3, GPHN*), thereby leading to Moco deficiency (MoCD) and loss of the Moco dependent SO activity (Kohl et al., 2018). Typically, MoCD and ISOD patients present in the neonatal period with identical clinical phenotypes, including encephalopathy, intractable seizures, feeding difficulties, and movement abnormalities. Disease progression involves psychomotor retardation due to progressive cerebral atrophy and ventricular dilatation, often resulting in fatal outcome within their first years of life (Schwarz, 2016). The most prominent biochemical hallmark of MoCD and ISOD is the accumulation of SO_3_^2–^ and the sulfite-cysteine adduct S-sulfocysteine in patient urine or plasma (Mudd et al., 1967). However, the disorders are distinguishable on the biochemical level due to the accumulation of xanthine and hypoxanthine, substrates of xanthine oxidase, another Moco enzyme, and diminished levels of uric acid in MoCD, but not ISOD (Schwarz et al., 2009; Schwahn et al., 2015).

Recently, we discovered a new mechanism underlying the neurodegeneration in SO deficiencies as we found that S-sulfocysteine can act as an NMDA receptor agonist and therefore leads to excitotoxic neuronal cell death (Kumar et al., 2017). SO_3_^2–^ toxicity has also been investigated in multiple studies, which indicate a disturbance of mitochondrial functions in neurons and kidney cells (Vincent et al., 2004; Zhang et al., 2004; Grings et al., 2014). In particular, exogenous SO_3_^2–^ decreased intracellular ATP levels, impaired cellular respiration and inhibited the mitochondrial enzymes glutamate dehydrogenase and malate dehydrogenase. Recently, impairment of mitochondrial respiration has also been shown in various patient cell lines with defects in cysteine catabolism (Grings et al., 2019).

Mitochondria are dynamic organelles constantly undergoing the processes of fusion and fission (Wai and Langer, 2016). The balance of fusion and fission can be influenced in either direction by a multitude of different factors including cellular respiration, ROS formation or the metabolic state of the cell, causing mitochondria to become hypertubular or fragmented. While the effect of SO_3_^2–^ on cellular bioenergetics and mitochondrial enzymes has been investigated before, the influence of SO_3_^2–^ on mitochondrial dynamics and morphology remains to be determined. In this study, we used SO deficient (*Suox*^–/–^) mouse embryonic fibroblast (MEF) cells to characterize mitochondrial morphology and function in SO deficiencies to shed light on the role of mitochondria in the disease pathology of ISOD and MoCD.

## Materials and Methods

### Cell Culture

Mouse embryonic fibroblast cells were harvested from heterozygous breedings of *Suox*^±^ mice (Kohl et al., unpublished) as described previously (Xu, 2005). In brief, embryos were removed at E13.5 after timed matings and dissected from the uterus. Fetuses were then minced into fine pieces and incubated in 0.25% trypsin-EDTA at 4°C overnight. On the next day, the suspension was heated for 30 min in a 37°C water bath. The digested tissue was then further broken down into a cell suspension by vigorous pipetting in culture medium. MEF cells were cultivated in Dulbecco’s Modified Eagle Medium (DMEM) (Pan Biotech), supplemented with 2 mM glutamine (Gibco by Life Technologies) and 10% Fetal Bovine Serum (FBS) (Pan Biotech; Origin: South Africa). For galactose treatment, cells were cultured in glucose-free DMEM (Pan Biotech, P04-01548S1) supplemented with 10 mM galactose or equal amounts of glucose as control.

Human fibroblasts were extracted from juvenile foreskin. Control fibroblasts were purchased from PromoCell (C-12300). Patient fibroblasts were kindly provided by J. Reiss (Institute for Human Genetics, University of Göttingen, Germany). *GPHN* deficient fibroblasts were first described in Reiss et al. (2001). *MOCS1*-deficient fibroblast were part of an earlier study (Reiss and Hahnewald, 2011). *MOCS2* deficient fibroblasts were characterized in Hahnewald et al. (2006). Fibroblasts were kept in RPMI 1640 medium (Pan Biotech) supplemented with 2 mM glutamine (Gibco by Life Technologies) and 10% FBS (Pan Biotech; Origin: South Africa). All cells were cultured at 37°C and 5% CO_2_.

### Mouse Keeping

All animals were kept and bred in accordance with European, national and institutional guidelines and protocols were approved by local government authorities (Landesamt für Natur, Umwelt und Verbraucherschutz Nordrhein-Westfalen, Germany; reference 84-02.04.2014.A372). Mice were kept under a 12 h light cycle and provided with regular chow diet and water *ad libitum*. For the generation of homozygous *Suox*^–/–^ mice, heterozygous mice of at least 2 months of age were kept in 1:1 (male:female) breedings.

### Mitochondrial Morphology Analysis

Mitochondrial morphology in MEF cells and patient fibroblasts was visualized using MitoTracker^TM^ Red CMXRos (Invitrogen, United States). The cells were seeded onto sterile cover slips the day prior to the experiment. Cells were incubated in 200 nM MitoTracker^TM^ Red CMXRos diluted in fresh DMEM for 30 min at 37°C. For SO_3_^2–^ treatments, the respective SO_3_^2–^ concentration was added to the medium 30 min prior to the MitoTracker and incubated at 37°C. Afterward, the cells were washed carefully with PBS before fixation in 4% PFA for 15 min at 4°C. Remaining PFA was removed by three washing steps with PBS (3 × 5 min). Finally, cover slips were mounted on slides with Mowiol/DABCO (Carl Roth). Images were acquired using a Nikon A1 confocal laser scanning microscope. Images were processed using ImageJ software.

### Mitochondrial Membrane Potential

Mitochondrial membrane potential was assessed as described previously with minor modifications (Verburg and Hollenbeck, 2008). In short, a 5 μM stock of tetramethylrhodamine methyl ester (TMRM, Invitrogen) in DMSO was diluted into DMEM medium to a final concentration of 20 nM. Cells were incubated in 20 nM TMRM for 20 min at 37°C. As a control, cells were co-stained with 200 nM MitoTracker^TM^ Deep Red (which accumulates in mitochondria independent of their membrane potential) for 20 min at 37°C. Images were acquired with a Nikon A1R confocal microscope. Pixel intensity for the TMRM was subsequently quantified using ImageJ (ImageJ, RRID:SCR_003070) and normalized by the respective intensity of the MitoTracker^TM^ Deep Red staining.

### Mitochondrial Motility and Content

The day prior to the experiment, cells were seeded onto a Nunc^TM^ glass-bottom dish (Thermo Scientific, United States). Mitochondria were visualized via incubation with 200 nM MitoTracker^TM^ Deep Red (diluted in DMEM) for 20 min at 37°C. Mitochondrial motility was analyzed via life cell imaging using a Nikon A1R confocal microscope with environmental chamber. Time-lapse videos were taken over 5 min at 37°C and 5% CO_2_. Mitochondrial motility and content were quantified after creating binary images with the software ImageJ (ImageJ, RRID:SCR_003070) as described in Koopman et al. (2006). In brief, maximum intensity projections of image stacks were converted to 8-bit formats and then subjected to a white top-hat filter (MorphoLibJ plugin) before a final thresholding step. Finally, the amount of moved pixels of selected mitochondria between the first and last timepoint (always 5 min apart) were assessed using the XOR function of the Image Calculator.

### Transmission Electron Microscopy

For Transmission electron microscopy (TEM), MEF cells were cultured on aclar foil and fixed with pre-warmed fixative solution (2% glutaraldehyde, 2.5% sucrose, 3 mM CaCl_2_, 100 Mm HEPES, pH 7.3) at RT for 30 min and 4°C for another 30 min. Afterward, fixed cells were washed with 0.1 M sodium cacodylate buffer, incubated with 1% OsO_4_, 1.25% Sucrose, 10 mg/ml Potassium ferrocyanide in 0.1 M Cacodylate buffer for 1 h on ice, and washed three times with 0.1 M Cacodylate buffer. Subsequently, cells were dehydrated using ascending ethanol series (50, 70, 90, 3 × 100) for 7 min each at 4°C. After that, cells were incubated with ascending EPON series at 4°C: EPON with ethanol (1 + 1) for 1 h, EPON with ethanol (3 + 1) for 2 h, EPON alone ON, 2 × 2 h with fresh EPON at RT and finally embedded for 48–72 h at 62°C. Ultrathin sections of 70 nm were cut with a diamond knife using an ultramicrotome (Leica, UC7) and stained with uranyl acetate for 15 min at 37°C and lead nitrate solution for 4 min. Electron micrographs were taken with a JEM-2100 Plus Electron Microscope (JEOL). Camera OneView 4K 16 bit (Gatan), and software DigitalMicrograph (Gatan).

### ATP Measurement

Intracellular ATP levels in MEF cells were quantified using the CellTiter-Glo^®^ Luminescent Cell Viability Assay (Promega, United States). In short, 8 × 10^4^ cells per well were seeded in 6-well plates the day prior to the experiment. The cells were incubated in glucose- or galactose containing medium for 1 h. They were then lysed in 150 μl RIPA buffer (Sigma). After centrifugation for 20 min at 4°C and 13,000 *g*, 50 μl of the lysate was added to 50 μl CellTiter-Glo^®^ Reagent in a 96-well. The plate was placed on an orbital shaker for 2 min to mix the contents before a 10 min incubation at room temperature. Luminescence was recorded using an EnVision Multimode Plate Reader (PerkinElmer, United States). ATP levels were quantified by comparison to a standard curve and normalized to the protein concentration in the respective sample.

### Cell Viability Assay

Cell viability after incubation in glucose- or galactose containing medium was assessed by the MTT assay as described previously (Kumar et al., 2017). In brief, 1 × 10^4^ cells/well were seeded onto a 96-well and grown for 24 h. The next day, the medium was exchanged for DMEM containing either 10 mM glucose or galactose as a carbon source. After 24 h, the old medium was exchanged with fresh phenol red- free DMEM containing 100 μM (3-(4,5-dimethylthiazol-2-yl)-2,5-diphyenyltetrazolium bromide) (MTT). After a 4 h incubation at 37°C in the dark, the MTT solution was exchanged for 100% DMSO. The plate was then placed in a shaker for 10–30 min at 37°C until all cells were solubilized. Finally, the absorption was measured at 570 nm (reference 650 nm) in well plate reader (Tecan Spark).

### Determination of Protein Concentration

The concentration of proteins from cell extracts was determined by means of the Bradford assay. Therefore, 190 μl diluted Bradford solution (1/5 dilution) was incubated with 10 μl of protein solution (respective dilution) for 20 min and the absorption change at 595 nm was measured using a well plate reader (BioTek, Germany). The determined absorption change was then compared to that of standard proteins with known concentration in order to determine the protein concentration of unknown solutions.

### Western Blot Analysis

Western blotting was performed on crude protein extracts from MEF cells lysed in 100 mM Tris/Ac, pH 8.0. Protein concentration was adjusted accordingly after determination with the Bradford assay. Unless otherwise indicated, 30 μg of protein lysate were separated by SDS-PAGE and immunoblotted using a standard semi-dry blotting protocol onto PVDF membranes. SO protein levels were visualized using a monoclonal anti-SUOX antibody (Sigma-Aldrich Cat# WH0006821M1, RRID:AB_1843810) in conjunction with a HRP-coupled anti-mouse secondary antibody (Santa Cruz Biotechnology, United States Cat# sc-2055, RRID:AB_631738). Vinculin was detected as loading control using a polyclonal anti-vinculin (H-300) antibody (Santa Cruz Biotechnology, United States Cat# sc-5573, RRID:AB_2214507) together with a HRP-coupled anti-rabbit secondary antibody (Santa Cruz Biotechnology, United States Cat# sc-2054, RRID:AB_631748). Signals were detected using chemiluminescent substrates (Thermo Fisher Scientific, United States, #34580) and a Bio-Rad ChemiDoc XRS + system.

## Results

### *Suox*^–/–^ MEFs Show Mitochondrial Impairment

**FIGURE 1:**
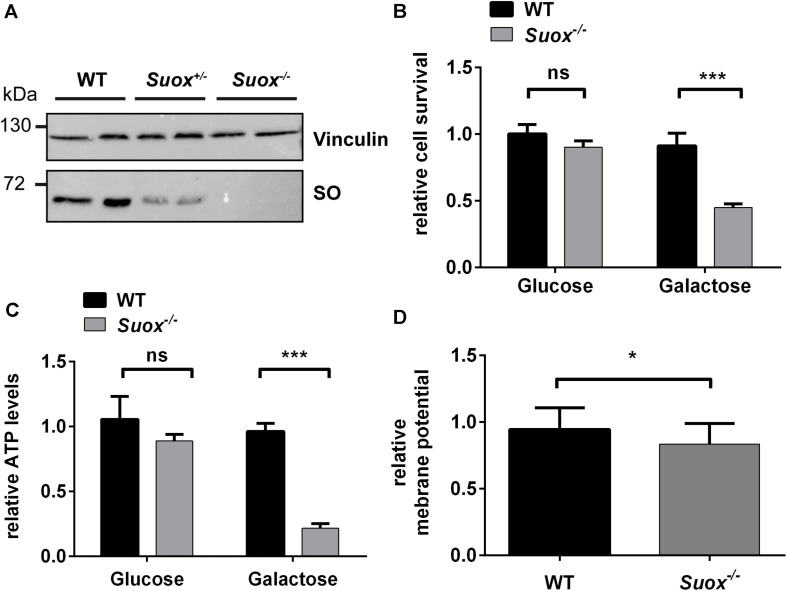
Mitochondrial deficiency in *Suox*^–/–^ MEFs. **(A)** Sulfite oxidase (SO) protein expression in WT, *Suox*^±^ and *Suox*^–/–^ MEFs. Vinculin was used as a loading control. **(B)** Determination of cell survival of WT and *Suox*^–/–^ MEFs after a 24 h incubation in glucose- or galactose containing DMEM medium (*n* = 6). Cell viability was assessed using the MTT assay (Promega). Values were normalized to WT grown in glucose medium. **(C)** Measurement of cellular ATP levels of WT and *Suox*^–/–^ MEFs after a 24 h incubation in glucose- or galactose containing DMEM (*n* = 3). Intracellular ATP levels were quantified using the CellTiter-Glo Luminescent Cell Viability Assay (Promega). Values were normalized to WT grown in glucose medium. **(D)** Determination of mitochondrial membrane potential in WT and *Suox*^–/–^ MEFs (*n* = 3). Mitochondrial membrane potential was assessed by staining with 20 nM TMRM (membrane potential-dependent). The pixel intensity was quantified relative to 200 nM MitoTracker Deep Red staining (membrane potential independent). Values were normalized to WT measurement. **(B–D)**: Error bars indicate standard deviation. Student’s *t* test was performed as indicated. *p* value: *** <0.001; ** <0.01; * <0.05; ns > 0.05.

Since previous studies indicate that SO_3_^2–^ impacts mitochondrial function (Vincent et al., 2004; Zhang et al., 2004; Grings et al., 2014), we first tested whether SO-deficient MEF cells (Figure 1A) generated from *Suox*^–/–^ mice (Kohl et al. unpublished results) exhibited signs of mitochondrial damage. We cultured WT and *Suox*^–/–^ MEFs in either glucose- or galactose-containing medium, thereby forcing the cells to rely on oxidative phosphorylation (OXPHOS) as the main energy source in absence of glucose (galactose-containing medium). After growing the cells for 24 h, we analyzed cell survival using the MTT assay (Figure 1B). While the absence of glucose had no effect on the viability of WT cells, survival of *Suox*^–/–^ cells was reduced to less than 50% in galactose, which indicates mitochondrial damage. We then measured intracellular ATP levels under the same conditions and again found no effect on WT MEFs, while *Suox*^–/–^ cells exhibited a severe reduction of ATP levels in galactose-containing medium (∼20% of control), thus showing that *Suox*^–/–^ MEFs are impaired in their ability to produce ATP via OXPHOS (Figure 1C). An essential component of OXPHOS is the generation of a proton gradient by different complexes of the respiratory chain (I, III, and IV). We therefore analyzed the mitochondrial membrane potential in WT and *Suox*^–/–^ cells via TMRM staining and found that *Suox*^–/–^ cells have a mild, but significant reduction in their membrane potential relative to WT (Figure 1D).

### Mitochondrial Morphology Is Altered in *Suox*^–/–^ MEFs

**FIGURE 2:**
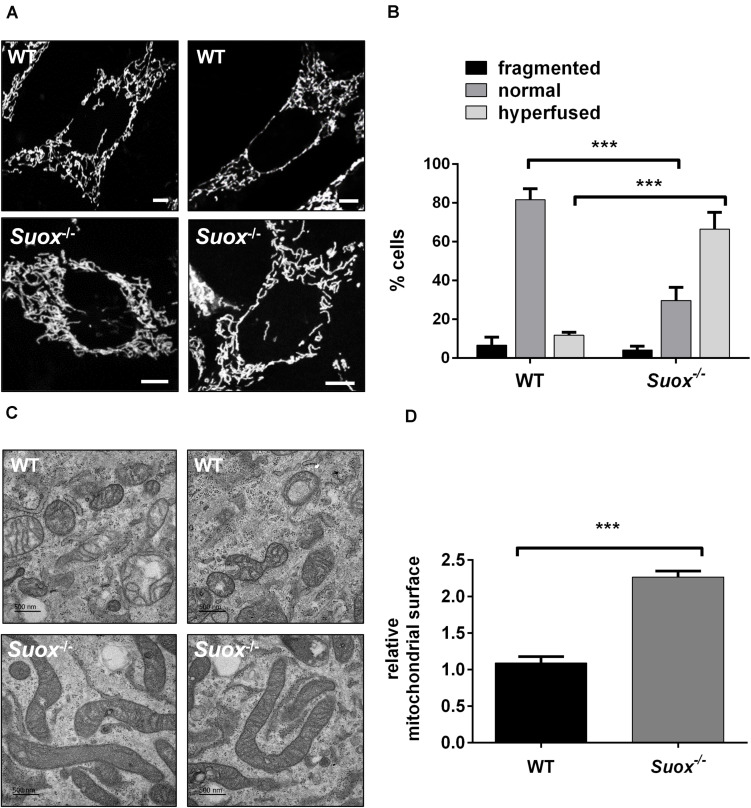
Mitochondrial morphology in *Suox*^–/–^ MEFs. **(A)** Visualization of the mitochondrial network in WT and *Suox*^–/–^ MEFs. Mitochondrial networks in WT and *Suox*^–/–^ MEFs were visualized by incubation in 200 nM MitoTracker Red CMXRos (Invitrogen) for 30 min at 37°C. *Suox*^–/–^ MEFs show a more interconnected and elongated mitochondrial network than WT cells. Scale-bar represents 10 μm. **(B)** Quantification of mitochondrial network phenotypes in WT and *Suox*^–/–^ MEFs presented in panel **(A)**. 100 cells per genotype were categorized according to their mitochondrial network as either fragmented (disturbed, dot-like network, almost no tubules present), normal (both tubules and dot-like mitochondria present) or hyperfused (highly interconnected tubules, no dot-like mitochondria present) (*n* = 3). Error bars indicate standard deviation. Ordinary two-way ANOVA with Tukey’s *post hoc* test for pairwise comparisons was performed as indicated. *p* value: *** <0.001; ** <0.01; * <0.05; ns > 0.05. **(C)** Mitochondria captured from WT and *Suox*^–/–^ MEFs via TEM. *Suox*^–/–^ MEFs show highly elongated mitochondria compared to WT cells. Scale bar represents 500 nm. **(D)** Quantification of the mitochondrial surface area in WT and *Suox*^–/–^ MEFs based on TEM pictures presented in **(C)**. The surfaces of 50 individual mitochondria were measured with ImageJ and normalized to WT values (*n* = 3). Error bars indicate standard deviation. Student’s *t* test was performed as indicated. *p* value: *** <0.001; ** <0.01; * <0.05; ns > 0.05.

Next, we visualized mitochondrial morphology in WT and *Suox*^–/–^cells after staining with MitoTracker Red CMXRos (Figure 2A). We observed that mitochondria in *Suox*^–/–^ MEFs formed a tightly interconnected and hypertubular network distinct from WT MEF cells. We quantified 100 cells per genotype and categorized the mitochondrial network as either fragmented (disturbed, dot-like network, almost no tubules present) normal (both tubules and dot-like mitochondria present) or hyperfused (highly interconnected tubules, no dot-like mitochondria present). We found that for WT cells 81% had a normal mitochondrial network, while 6% displayed fragmented mitochondria, and 13% were hyperfused (Figure 2B). In contrast, only 30% of the *Suox*^–/–^ cells had a normal mitochondrial network, while 4% had a fragmented network, and 66% had a hyperfused network. To complement these light microscopic studies, we performed TEM and again found the mitochondria in the KO cells to be abnormally elongated compared to WT (Figure 2C). Quantification of the cross-sectional area of 50 individual mitochondria confirmed that mitochondria from *Suox*^–/–^ MEFs were on average more than twice as large than WT mitochondria (Figure 2D). Finally, TEM revealed that the elongated mitochondria also showed a much more electron dense matrix structure than WT mitochondria.

### *Suox*^–/–^ Mitochondria Are Less Motile Than WT

**FIGURE 3:**
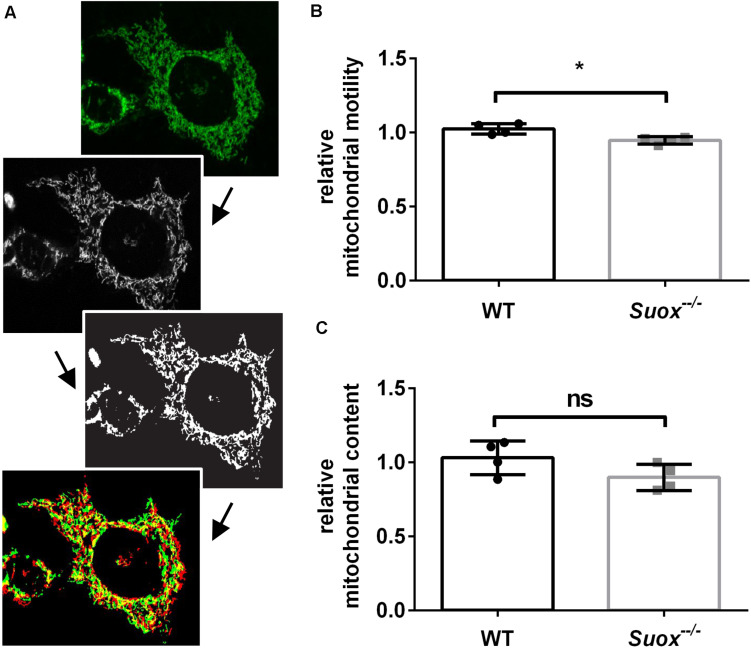
Mitochondrial motility and content in *Suox*^–/–^ MEFs. **(A)** Workflow of mitochondrial motility analysis using ImageJ. Starting from the left, maximum intensity projections of image stacks **(first picture)** were converted to 8-bit formats **(second picture)** and then subjected to a white top-hat filter (MorphoLibJ plugin), before a final thresholding step **(third picture)**. Finally, the amount of moved pixels of selected mitochondria **(last picture)** between the first (green) and last (red) timepoint (always 5 min apart) were assessed using the XOR function of the Image Calculator with yellow pixel indicate overlapping pixels. **(B)** Quantification of mitochondrial motility as shown in **(A)**. Mitochondrial motility was analyzed via life cell imaging following incubation with 200 nM MitoTracker Deep Red (diluted in DMEM) for 20 min at 37°C. Values were normalized to WT. Error bars indicate standard deviation. Student’s *t* test was performed as indicated. *p* value: *** <0.001; ** <0.01; * <0.05; ns > 0.05. **(C)** Quantification of mitochondrial content. Mitochondria were visualized via incubation with 200 nM MitoTracker Deep Red (diluted in DMEM) for 20 min at 37°C. Total amount of pixels per cell was quantified using ImageJ. Values were normalized to WT. Error bars indicate standard deviation. Student’s *t* test was performed as indicated. *p* value: *** <0.001; ** <0.01; * <0.05; ns > 0.05.

Mitochondria have a high degree of motility, which enables them to distribute throughout the cytoplasm and facilitates both fusion and fission processes. To measure mitochondrial mobility, we acquired time-lapse videos of mitochondrial movements in an environmental chamber at 37°C over a period of 5 min. The first and last image of each video were colored red or green and overlaid. Yellow pixels represented mitochondria that remained stationary over the 5 min period, while red and green pixels were quantified as a proxy measure of mitochondrial motility (Yi et al., 2004; Figure 3A). Our quantification showed that *Suox*^–/–^ mitochondria moved less than WT mitochondria (Figure 3B). We also measured the total amount of mitochondria per cell and found no statistically significant difference, although *Suox*^–/–^ cells tend to have less mitochondrial content than WT cells (Figure 3C).

### Sulfite Treatment Leads to Abnormal Mitochondrial Morphology

**FIGURE 4:**
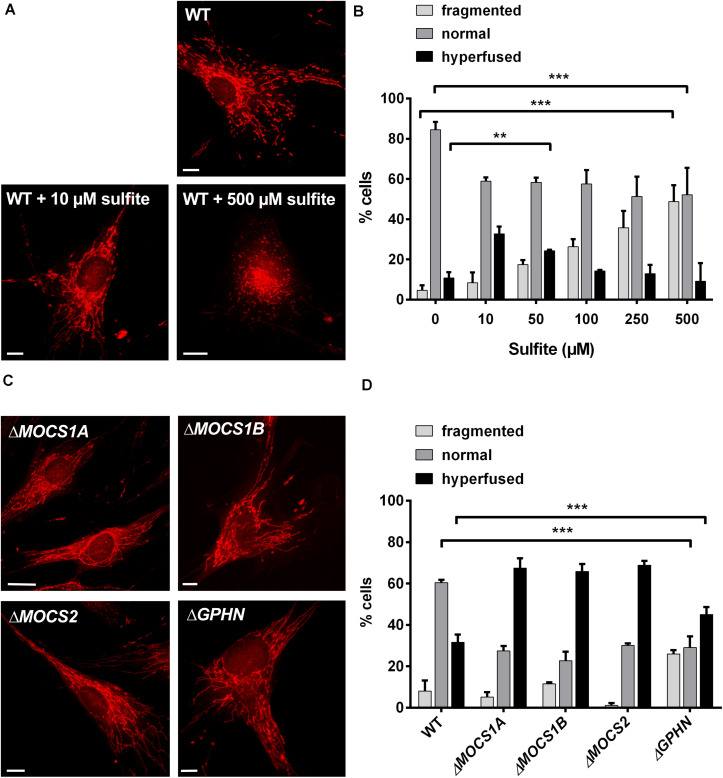
Influence of sulfite (SO_3_^2–^) on mitochondrial morphology. **(A)** Imaging of the mitochondrial network in human WT fibroblasts with and without SO_3_^2–^. Mitochondria in fibroblasts were visualized using 200 nM MitoTracker Red CMXRos (Invitrogen) for 30 min at 37°C. For SO_3_^2–^ treatments, the respective SO_3_^2–^ concentration was added to the medium 30 min prior to the MitoTracker and incubated at 37°C. While untreated WT cells show a normal mitochondrial network **(upper left)**, treatment with lower SO_3_^2–^ concentrations (10 μM) leads to a hypertubular network **(upper right picture)**. Higher SO_3_^2–^ concentrations (<500 μM) result in mitochondrial fragmentation **(lower left picture)**. Scale-bar represents 10 μm. **(B)** Quantification of mitochondrial network phenotypes in WT fibroblasts after treatment with different SO_3_^2–^ concentrations as presented in **(A)**. 100 cells per genotype were categorized according to their mitochondrial network as either fragmented (disturbed, dot-like network, almost no tubules present), normal (both tubules and dot-like mitochondria present) or hyperfused (highly interconnected tubules, no dot-like mitochondria present, length) (*n* = 3). Error bars indicate standard deviation. Ordinary two-way ANOVA with Tukey’s *post hoc* test for pairwise comparisons was performed as indicated. *p* value: *** <0.001; ** <0.01; * <0.05; ns > 0.05. **(C)** Imaging of the mitochondrial network in fibroblasts from different MoCD patients. Mitochondria in different patient fibroblasts were visualized using 200 nM MitoTracker Red CMXRos (Invitrogen) for 30 min at 37°C. All cell lines presented strongly interconnected and hyperfused mitochondria. Scale-bar represents 10 μm. **(D)** Quantification of mitochondrial network phenotypes in fibroblasts from different MoCD patients presented in **(C)**. 100 cells per genotype were categorized according to their mitochondrial network as either fragmented (disturbed, dot-like network, almost no tubules present), normal (both tubules and dot-like mitochondria present) or hyperfused (highly interconnected tubules, no dot-like mitochondria present) (*n* = 3). Error bars indicate standard deviation. Ordinary two-way ANOVA with Tukey’s *post hoc* test for pairwise comparisons was performed as indicated. *p* value: *** <0.001; ** <0.01; * <0.05; ns > 0.05.

We reasoned that the alterations in mitochondrial morphology observed in *Suox*^–/–^ cells could be a direct result of intracellular SO_3_^2–^ accumulation. To test whether SO_3_^2–^ affects mitochondrial morphology, we treated human WT fibroblasts with escalating doses of SO_3_^2–^ concentrations and assessed for changes in mitochondrial morphology after 1 h of treatment (Figure 4A). Addition of 10 or 50 μM SO_3_^2–^ led to an increase in the percentage of cells with hyperfused mitochondria (Figure 4B). While we found that ∼11% of untreated WT cells were hyperfused, addition of 10 or 50 μM SO_3_^2–^ resulted in ∼32% and 23% hyperfused cells, respectively. We also observed that treatment with higher SO_3_^2–^ concentrations lead to a higher percentage of fragmented mitochondria. Around 50% of the cells displayed fragmented mitochondria after treatment with 500 μM SO_3_^2–^, whereas untreated cells showed fragmented mitochondria in ∼4% of all analyzed cells. In turn, the number of cells categorized to harbor a normal mitochondrial network were significantly reduced in all SO_3_^2–^-treated cells, regardless of the respective concentration. While 84% of untreated cells had a normal mitochondrial network, this percentage was reduced to ∼57% for SO_3_^2–^ concentrations up to 100 μM and ∼50% for 250 and 500 μM SO_3_^2–^ treatments. These data show that SO_3_^2–^ directly influences mitochondrial morphology in a dose-dependent manner.

### MoCD Patient Derived Fibroblasts Recapitulate the Mitochondrial Phenotype of *Suox*^–/–^ MEF Cells

Finally, we analyzed mitochondrial morphology in fibroblasts derived from various MoCD patients that are impaired in different genes important for Moco biosynthesis, namely *MOCS1A*, *MOCS1B*, *MOCS2*, and *GPHN* (Figure 4C). We found that cell lines derived from patients with disease causing mutations in *MOCS1* or *MOCS2*∼70% contained a hyperfused mitochondrial network, whereas this was only the case for 30% of the cells for WT fibroblasts (Figure 4D). In cells derived from patients with disease causing mutations in the *GPHN* gene, 45% were characterized as hyperfused. In turn, ∼60% of WT cells displayed a normal mitochondrial network, while this phenotype could only be found in ∼25–30% of all patient cells regardless of the affected gene. The percentage of cells with a fragmented mitochondrial network varied between 1% and 10% for the *MOCS1/2* and WT cell lines. Remarkably, only 1% of the cells deficient in *MOCS2* were fragmented, whereas 25% of the cells affected in *GPHN* showed fragmented mitochondria. In summary, these data confirm a disturbed mitochondrial network in different patient cell lines of MoCD that can collectively be traced back to SO_3_^2–^ accumulation.

## Discussion

Sulfite oxidase is a mitochondrial enzyme that is vital for the detoxification of SO_3_^2–^. Inactivation of SO, in either ISOD or MoCD, leads to severe neurodegeneration, often with lethal outcome. While mitochondrial impairment has been observed following SO_3_^2–^ treatment of different model systems (Vincent et al., 2004; Zhang et al., 2004; Grings et al., 2013), the underlying mechanism and role of mitochondrial damage within the context of SO deficiency remains poorly understood. In this study, we show that mitochondria in *Suox*^–/–^ and patient fibroblasts are abnormally elongated and interconnected. Application of exogenous SO_3_^2–^ on WT fibroblasts induced similar morphological changes in the mitochondrial network, suggesting that SO_3_^2–^ accumulation in SO-deficient cells directly mediates alterations in mitochondrial function. This pathomechanism provides a new concept underlying SO_3_^2–^ toxicity in the SO deficiencies.

We found that cellular viability and ATP production under galactose treatment was markedly reduced in *Suox*^–/–^ MEFs, indicating that the mitochondria in those cells are dysfunctional and thus unable to produce sufficient ATP via OXPHOS. In line with this, one of the earliest identified toxic effects of SO_3_^2–^ was a dose-dependent reduction of total cellular ATP levels (Zhang et al., 2004). Moreover, direct treatment of SO_3_^2–^ also decreased ATP production from mitochondria isolated from rat renal epithelial cells (Vincent et al., 2004). Therefore, it is likely that elevated SO_3_^2–^ levels, due to the lack of SO, lead to the inhibition of mitochondrial ATP production and reduced viability upon longer cultivation in galactose.

In a recent study, Grings et al. (2019) reported that *MOCS1* patient fibroblasts display altered protein levels of mitofusins 1 and 2, indicating a disturbance of mitochondrial dynamics. We analyzed the mitochondrial network in different cell culture models of SO deficiency and found that the mitochondria were hyperfused in the vast majority of SO-deficient cells. Furthermore, treatment of WT cells with low SO_3_^2–^ concentrations (up to 40 μM) resulted in similar morphological changes, suggesting that the observed abnormalities are directly mediated by SO_3_^2–^. Hyperfusion is known as a typical response to mild mitochondrial stress, for example nutrient starvation, as it allows to separate mitochondria within one cell to exchange their contents and thereby help to “recover” individual damaged mitochondria (Rugarli et al., 2012). However, treatment of WT cells with high SO_3_^2–^ concentrations (more than 50 μM) lead to increased mitochondrial fragmentation, indicating that at this point, the mitochondrial damage becomes too severe to be repaired. Mitochondrial fragmentation can be induced by various different forms of stress and allows the removal of terminally damaged mitochondria by mitophagy (Rugarli et al., 2012). In particular, inhibition of the mitochondrial enzymes malate dehydrogenase and glutamate dehydrogenase has been shown for SO_3_^2–^ concentrations of 100 μM and higher (Zhang et al., 2004). Inhibition of the central metabolic enzyme glutamate dehydrogenase could lead to a decreased metabolic flux through the TCA cycle, which might further contribute to the reduced ATP production, thereby inducing fragmentation.

Hyperfusion of mitochondria may be induced by an increase in fusion or a decrease in fission processes. We detected a decrease in mitochondrial motility in *Suox*^–/–^ cells, which might suggest a decrease in fission. Correct function of Drp1, a GTPase that mediates mitochondrial fission, is regulated by a number of different mechanisms, such as recruitment to the outer mitochondrial membrane by recruiting factors as well as post-translational modifications such as phosphorylation or nitrosylation (Suárez-Rivero et al., 2016). Following our initial findings, further studies are needed to elucidate the exact mechanism of how SO_3_^2–^ alters mitochondrial dynamics.

The most affected organ in the SO deficiencies is the nervous system. Interestingly, this is also the case in many other disorders of disrupted mitochondrial dynamics. Both Drp1 and mitofusin-2 are required for neuronal development and dysregulation of either protein is associated with a neurological disorder (Suárez-Rivero et al., 2016). For example, mitofusin-2 is necessary for cerebellar development in mice (Chen et al., 2007), and dominant negative variants of mitofusin-2 are associated with Charcot–Marie–Tooth disease type 2A, a length dependent peripheral neuropathy in man (Kijima et al., 2005). Drp1 on the other hand has been associated with multiple disorders, including Huntington’s disease, amyotrophic lateral sclerosis, and Parkinson’s disease (Song et al., 2011, 2013; Wang et al., 2011).

Among the group of mitochondrial disorders, there is another disease of sulfur metabolism, called ethylmalonic encephalopathy or ETHE1 deficiency (Tiranti et al., 2004). Similar to SO deficiencies, patients present with early onset progressive neurological degeneration, psychomotor retardation and excretion of ethylmalonic acid in urine, resulting in death in the first few years of life (Tiranti et al., 2004). ETHE1 patients accumulate high levels of H_2_S and thiosulfate in biological fluids and tissues, the latter also being elevated in ISOD and MoCD. Intriguingly, H_2_S has been reported to interfere with cellular respiration via inhibition of cytochrome c oxidase (Tiranti et al., 2009). First attempts have recently been made to improve mitochondrial respiratory function in fibroblasts from ETHE1 and MOCS1 patients by treating cells with the mitochondria-targeted antioxidant JP4-039 (Grings et al., 2019). While these results are promising, future studies should aim for further dissection of the underlying molecular mechanisms that primarily drive mitochondrial pathology in neuronal tissues of SO deficiency models in order to provide new therapy options. In summary, we show here that mitochondrial dynamics are impaired by SO_3_^2–^ and dysregulated in SO deficiencies, thereby providing further evidence that SO deficiencies join the large and diverse family of mitochondrial disorders.

## Data Availability Statement

The raw data supporting the conclusions of this article will be made available by the authors, without undue reservation.

## Ethics Statement

All animals were kept and bred in accordance with European, national and institutional guidelines and protocols were approved by local government authorities (Landesamt für Natur, Umwelt und Verbraucherschutz Nordrhein-Westfalen, Germany; reference 84-02.04.2014.A372).

## Author Contributions

A-TM, JR, and GS: study design. JK: providing of study materials. A-TM, JR, AM, and GS: data generation, analysis, and interpretation. A-TM, AM, and GS: manuscript writing. All authors contributed to the article and approved the submitted version.

## Conflict of Interest

The authors declare that the research was conducted in the absence of any commercial or financial relationships that could be construed as a potential conflict of interest.
